# Fungal and Bacterial Co-infection of Supraglottis in an Immunocompetent Patient Secondary to Herbal Medicine

**DOI:** 10.7759/cureus.26181

**Published:** 2022-06-21

**Authors:** Muhammad Sallehin Zulkaflay, Marina Mat Baki

**Affiliations:** 1 Otolaryngology - Head and Neck Surgery, Universiti Kebangsaan Malaysia Medical Centre, Kuala Lumpur, MYS; 2 Otolaryngology - Head and Neck Surgery, Hospital Melaka, Melaka, MYS

**Keywords:** herbal medicine, immunocompetent, supraglottitis, co-infection, bacterial, fungal

## Abstract

Fungal infections are among the major infections among immunocompromised patients. They are becoming more common with the widespread use of antibiotics, steroid therapy, and the increasing number of immunocompromised patients. However, the incidence of laryngeal fungal and bacterial co-infection has rarely been reported. As same as laryngeal fungal infection, it mimics other types of laryngeal disease such as gastroesophageal reflux disease, granulomatous disease, and malignant lesions. There is a high likelihood of misdiagnosis, leading to delayed treatment and morbidity from fungal and bacterial co-infection of the larynx. A high index of suspicion is required to make the diagnosis and one should look for evidence of immunosuppression and predisposing factors to local mucosal barrier impairment. However, herbal medicine is a rare cause. We present a case of fungal and bacterial co-infection of supraglottis in an immunocompetent patient secondary to herbal medicine.

## Introduction

Opportunistic laryngeal infection is primarily caused by pathogens that are normally present in the upper respiratory tract of the normal population which take advantage of a locally or systemically compromised immune system to invade and proliferate in the tract and becomes pathogenic. The microbial community of the respiratory tract is diverse and harbors many opportunistic pathogens. Candida, which is widespread in the natural world, is found to be both the most common opportunistic organism colonizing the respiratory tract and the causative agent of opportunistic laryngeal infection, namely fungal laryngitis [1]. Bacterial opportunistic respiratory tract infection is common but rarely reported in cases of laryngitis. Among the most frequent bacterial pathogens are *Streptococcus pneumoniae, Haemophilus influenzae,* and *Staphylococcus aureus* [2].

Co-infection of fungal and bacterial supraglottitis is a rare clinical entity. Immunocompromised patients with diabetes, AIDS, leukemia, aplastic anemia, and long-term use of steroids as well as patients with certain medical conditions like inhaled steroid therapy, laryngopharyngeal reflux disease, prior radiotherapy, and prolonged antibiotic use are at high risk of infection [3]. Herbal medicine is known to have immunomodulatory properties [4], but there are no reports found so far on herbal medicine causing opportunistic infection. Hence, we present an extremely rare case of mixed fungal and bacterial supraglottitis that also involves the hypopharynx in a healthy individual in which herbal medicine is the single predisposing factor found. The management included proper identification and eradication of the organisms with antimicrobials, followed by complete cessation of the sole predisposing agent.

## Case presentation

**Figure 1:**
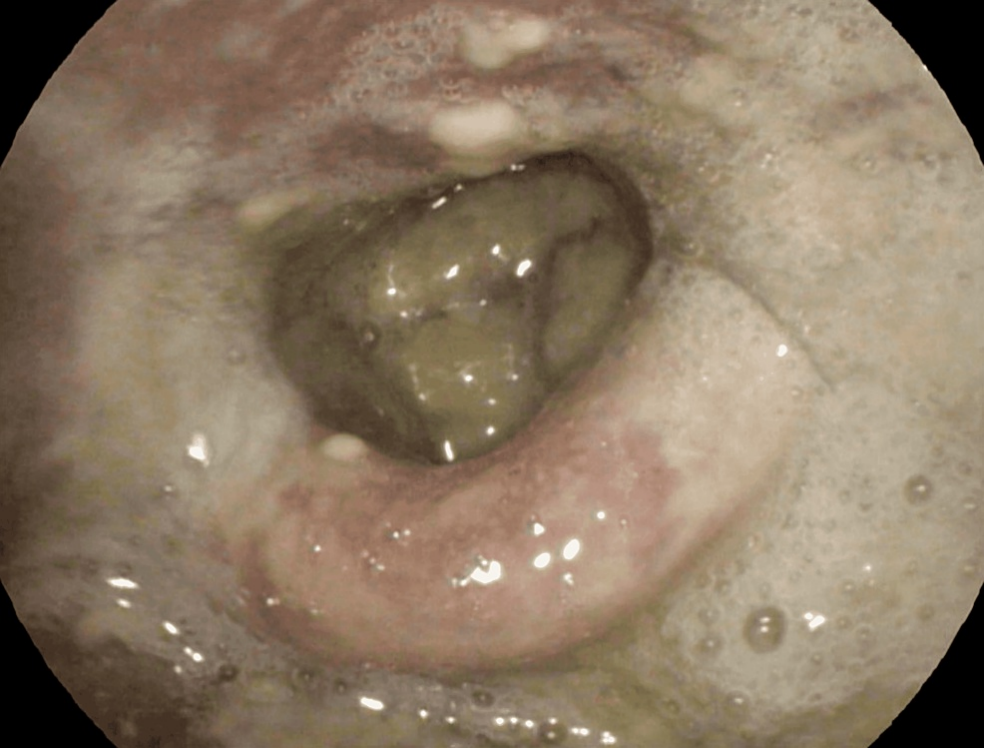
Supraglottitis characterized by edematous bilateral arytenoid and epiglottis with yellowish plaque extending to post cricoid area and aryepiglottic fold

An 84-year-old male, a chronic smoker with underlying hypertension, dyslipidemia, and a history of minor stroke 3 years before, presented with dysphagia and hoarseness associated with yellowish sputum for the past 6 months. The dysphagia was described as difficulty in swallowing solid food and the presence of a lump sensation in the throat. Neither of the symptoms has worsened over time. He denied any constitutional symptoms, shortness of breath, chest pain, or halitosis. Flexible laryngoscopy showed edematous bilateral arytenoids and epiglottis with the presence of yellowish plaque extending to post cricoid area and aryepiglottic fold and significant pooling of saliva (Figure 1). However, bilateral vocal folds were less affected. Other examinations including neck, nasal, and oral cavity were unremarkable.

A provisional diagnosis of tuberculous laryngitis with a differential diagnosis of fungal laryngitis was made. A sputum sample was collected and sent for tuberculosis workup and culture. The sputum was negative for tuberculosis but the culture grew* Candida albicans* and *Klebsiella pneumoniae,* hence a diagnosis of fungal laryngitis with bacterial co-infection was made. He was thoroughly screened for any conditions, including immunodeficiency status, diabetes, prolonged usage of immunosuppressive drugs, and previous radiotherapy, all of which were negative. The patient’s routine blood investigations and chest X-ray were normal. Upon further questioning, he admitted to taking herbal medicine which was composed of Curcumae xanthorrhizae rhizoma, Curcumae domesticae rhizoma, and Imperatae cylindricae rhizoma for the past 3 years on a daily basis. The reasons for him taking the herbal medicine were for good health and to prevent another recurrence of the minor stroke. Thus, we attributed his condition to his herbal medicine consumption as the predisposing factor.

**Figure 2:**
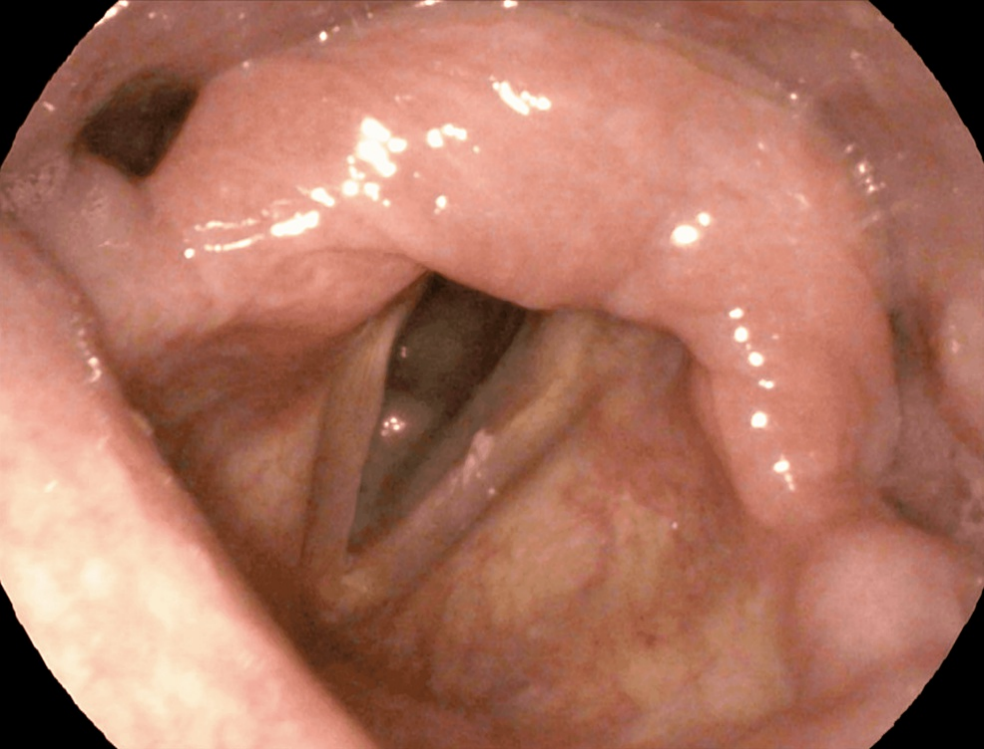
Laryngoscopy after completion of the antimicrobials and cessation of the herbal medicine

The patient was prescribed oral fluconazole 100 mg once daily and oral cefuroxime 500 mg twice daily and was instructed to stop consuming the herbal medicine. After 2 weeks, the patient showed a remarkable improvement after the full course of the antifungals was completed, and flexible laryngoscopy revealed a reduction of the lesions in the larynx (Figure 2).

Two months after his initial evaluation, the patient complained of a recurrence of symptoms. Upon further questioning, the patient had resumed the consumption of the herbal medicine for the same reason after the resolution of his symptoms. The flexible laryngoscopy showed similarities to the initial findings. The same antimicrobials were restarted and completed for 2 weeks, and the herbal medicine was discontinued. The patient has been well since then.

## Discussion

Although herbal medicines are widely considered to be of lower risk compared to synthetic drugs, they are not entirely free of potential toxicities and side effects. Studies have shown that herbal medicines have the capacity to suppress the immune response effects through induction of apoptosis or modulation of cell proliferation and cytokine secretion, and therefore, can have beneficial applications in some immune-mediated disorders, including autoimmune diseases [4]. On the other hand, misuse of herbal medicines might spell a catastrophe for patients and immunosuppression may ensue.

In our case, the main components of the herbal medicines used by the patient were Curcumae xanthorrhizae rhizoma and Curcumae domesticae rhizoma, in which the bioactive compound is curcuminoid. Curcuminoid is a potent anti-inflammatory that can alter the expression of various transcription factors, cell cycle proteins, and signal transducing kinases [4]. High doses of curcumin have been shown to irreversibly inhibit the development of cell-mediated cytotoxicity, lymphocyte proliferation, and the production of cytokines [5]. The effect of this compound on B-cell activation has also been studied. At low doses, curcumin could enhance the antibody response, whereas, at high doses, it has been shown to reduce B-cell proliferation [6].

Another notable constituent is Imperatae cylindricae rhizoma. Saponins and flavonoids are two of its important bioactive compounds. Saponin is a large family of amphiphilic glycosides of steroids and triterpenes. It has shown a significant decrease in the number of monocytes and granulocytes count in human whole blood and a decline in CD3 count and macrophage activation in an animal model with a dosage-dependent relationship [7]. Flavonoids, a group of naturally occurring polyphenolic compounds have also been shown to inhibit various transcriptional factors whose function modulates immune cell differentiation, proliferation, and activation and enhances regulatory T-cell generation [8]. All of this explains the immunosuppressive event in our patient, which was caused by the herbal medicine, thus causing opportunistic laryngeal infection.

This case reports fungal laryngitis with a bacterial co-infection. A study has shown that a secondary bacterial infection of respiratory tract could prevail due to airway fungal colonization [9]. The presence of *Candida albicans* in the tract could impair the immune response which could decrease the normal antibacterial function of the immune system permitting bacterial pathogens to cause infection instead of being cleared by the immune cells. *Klebsiella pneumoniae* has been considered an opportunistic pathogen that commonly colonizes mucosal surfaces in humans and typically causes infections in hospitalized or immunocompromised individuals [10].

As seen in our case as well, the clinical findings of hoarseness and dysphagia often bear a resemblance to granulomatous disease and gastroesophageal reflux disease. With a history of chronic smoking, one may think of the diagnosis of a malignancy in the upper aerodigestive tract. With all of the differential diagnoses possible, if the diagnosis of fungal laryngitis is not proactively considered, it may be missed and eventually, inappropriate treatment and unwarranted surgical intervention may follow.

The role of biopsy is controversial. While some advocate biopsy of the lesion to establish the diagnosis, others reserve this for refractory cases or for cases that are highly suspicious of malignancy [11]. In our case, we initially did laryngeal culture alone and it was sufficient to confirm the diagnosis. However, close monitoring of symptoms and signs is warranted. Lack of clinical improvement after about two weeks of treatment should alert the clinicians to the need for direct laryngoscopy and biopsy. Other investigations should be directed at identifying risk factors. With the history of rampant use of herbal medicine, this case has led us to suspect the correct diagnosis. Subsequently, it guided us through the direction of the treatment.

## Conclusions

The diagnosis of opportunistic laryngeal infection in an immunocompetent patient is rare and yet, herbal medicine is not widely reported in the medical literature to cause such infections. It demands great attention on the presenting history and physical examination together with laboratory work-up in ruling out other conditions. The presence of dysphagia and hoarseness with chronic consumption of herbal medicine should raise the level of suspicion for the diagnosis, or otherwise, we might land ourselves in the wrong diagnosis and consequently, delayed treatment. Early diagnosis and treatment are crucial to prevent the spread and morbidity of fungal laryngitis. Recognition and elimination of the predisposing factor are of paramount importance as well.
